# Genetic Background of Metabolically Healthy and Unhealthy Obesity Phenotypes in Hungarian Adult Sample Population

**DOI:** 10.3390/ijms24065209

**Published:** 2023-03-08

**Authors:** Peter Piko, Erand Llanaj, Karoly Nagy, Roza Adany

**Affiliations:** 1 ELKH-DE Public Health Research Group, Department of Public Health and Epidemiology, Faculty of Medicine, University of Debrecen, 4032 Debrecen, Hungary; 2 Epidemiology and Surveillance Centre, Semmelweis University, 1085 Budapest, Hungary; 3 Department of Public Health and Epidemiology, Faculty of Medicine, University of Debrecen, 4032 Debrecen, Hungary; 4 Department of Public Health, Semmelweis University, 1089 Budapest, Hungary

**Keywords:** optimized genetic risk score, metabolically unhealthy obesity, metabolically healthy obesity, Hungarian population, lipid metabolism, glucose metabolism

## Abstract

A specific phenotypic variant of obesity is metabolically healthy (MHO), which is characterized by normal blood pressure and lipid and glucose profiles, in contrast to the metabolically unhealthy variant (MUO). The genetic causes underlying the differences between these phenotypes are not yet clear. This study aims to explore the differences between MHO and MUO and the contribution of genetic factors (single nucleotide polymorphisms—SNPs) in 398 Hungarian adults (81 MHO and 317 MUO). For this investigation, an optimized genetic risk score (oGRS) was calculated using 67 SNPs (related to obesity and to lipid and glucose metabolism). Nineteen SNPs were identified whose combined effect was strongly associated with an increased risk of MUO (OR = 1.77, *p* < 0.001). Four of them (rs10838687 in *MADD*, rs693 in *APOB*, rs1111875 in *HHEX*, and rs2000813 in *LIPG*) significantly increased the risk of MUO (OR = 1.76, *p* < 0.001). Genetic risk groups based on oGRS were significantly associated with the risk of developing MUO at a younger age. We have identified a cluster of SNPs that contribute to the development of the metabolically unhealthy phenotype among Hungarian adults suffering from obesity. Our findings emphasize the significance of considering the combined effect(s) of multiple genes and SNPs in ascertaining cardiometabolic risk in obesity in future genetic screening programs.

## 1. Introduction

Obesity is one of the alarmingly increasing global health challenges which presently affects more than a billion people worldwide [1], and it is disproportionately prevalent among vulnerable socioeconomic groups and ethnic minorities [2,3,4]. Its direct and indirect influence on non-communicable diseases (NCDs) are well-documented, with a special emphasis on the risk of type two diabetes (T2D), cardiovascular diseases (CVDs), and several types of cancer attributed to increased adiposity [5,6,7,8]. In addition to the fact that obesity increases the risk of certain diseases, the World Obesity Federation has also identified obesity itself as a progressive, chronic, relapsing disease [9]. This finding is supported by the fact that the pathophysiology of obesity is influenced by the interaction of environmental/lifestyle factors (such as an energy-dense diet and sedentary lifestyle) and genetic predisposition, which leads to increased body weight [9] and a positive energy balance.

Data from independent studies show that in a subgroup of obese individuals, no positive association between body mass index (BMI) and cardiometabolic risk was observed, and these individuals may be protected against obesity-related cardiometabolic diseases (CMD), or at least have a significantly lower risk of developing CMD [10] than expected. This is considered a metabolically healthy obesity (MHO) sub-phenotype, and despite the lack of a universal definition [11], such a phenotype refers to those being obese and yet having no cardiometabolic complications. Although there is no standardized definition of MHO, the following criteria have been proposed in addition to the diagnosis of obesity (BMI ≥ 30 kg/m^2^): fasting levels of serum glucose ≤ 6.1 mM (≤100 mg/dL); triacylglycerols (TAG) ≤ 1.7 mM (≤150 mg/dL); high-density lipoprotein (HDL) cholesterol > 1.0 mM (>40 mg/dL) in men or > 1.3 mM (>50 mg/dL) in women; systolic blood pressure (SBP) ≤130 mmHg; diastolic blood pressure (DBP) ≤ 85 mmHg; no drug treatment for dyslipidemia, diabetes, or hypertension; and no cardiovascular disease manifestation [12,13]. Accumulating evidence suggests that although people with metabolically healthy obesity could have a higher risk of all-cause mortality and cardiovascular events compared to metabolically healthy non-obese people, their risks are substantially lower compared to individuals with metabolically unhealthy obesity (MUO) [14,15].

The prevalence of metabolically healthy obesity is estimated to be somewhere between 10% and 51% depending on age and sex [12,16]. The proportion of MHO was higher in women and decreased with age, and slightly higher in groups of Asian origin compared to non-Asian origin [16].

Obesity is a complex trait influenced by genetic (polymorphism, epigenetic, and metagenomic factors) and non-genetic (environmental and lifestyle) factors and their interactions [17]. The elevated risk of obesity has been linked to individual single nucleotide polymorphisms (SNPs) in human genes encoding proteins such as adipocyte-, C1q-, and collagen domain-containing (*ADIPOQ*) fat mass- and obesity-associated gene (*FTO*), leptin (*LEP*), leptin receptor (*LEPR*), insulin-induced gene two (*INSIG2*), melanocortin four receptor (*MC4R*), proprotein convertase subtilisin/kexin type one (*PCSK1*), and peroxisome proliferator-activated receptor gamma (*PPARG*) [18,19].

Even though obesity is a hot topic in modern medicine and requires a multidisciplinary approach to its investigation, the genetic causes underlying the difference between metabolically healthy and unhealthy phenotypes are currently not well understood [20]. Presently, limited data exist with regard to a genetic predisposition to MHO/MUO on human samples. A publication by Berezina and colleagues in which 503 abnormally obese patients without cardiovascular disease were studied found that MHO and MUO patients did not differ in the distribution of the *LEP* gene genotypes G19G, G19A, and A19A and the Adiponectin (*AN*) gene genotypes G276G, G276T, and T276T [21]. Li and colleagues identified two SNPs (rs2237897 and rs2237892) in the Potassium Voltage-Gated Channel Subfamily Q Member one (*KCNQ1*) gene in Chinese children aged 6 to 18 years that influenced susceptibility to MHO in interaction with environmental and lifestyle factors. Sedaghati-khayat and colleagues identified four SNPs (rs1421085, rs1121980, rs1558902, and rs8050136) in the *FTO* gene that are significantly associated with MUO [22].

The impact of obesity on health [23,24] and the economy [25] is a growing burden that requires comprehensive knowledge to reduce. Since obesity (and its associated metabolic pathways) is significantly heritable and our knowledge of the reasons behind the difference between the MHO and MUO phenotypes is limited, it is worth conducting further research to identify the underlying genetic causes.

Hence, the present study aims to investigate the genetic background of the MHO and MUO phenotypes using obese samples from the Hungarian adult population, and to establish a genetic score that can be used to estimate the risk of MUO at an individual or a population level.

## 2. Results

### 2.1. Characteristics of the Obese Study Samples

After the exclusion of subjects with incomplete geno- and/or phenotype data, a total of 398 obese individuals (317 MUO and 81 MHO) remained in the database for the current analyses (Table 1).

Apart from expected differences in biomarkers’ values and the prevalence of medication use, MHO participants were younger compared to MUO, with no sex and education-specific differences. The statistical analyses were corrected for the age of the participants, thus avoiding any effects due to age differences.

### 2.2. The Best-Fitting Genetic Models by SNPs

Twenty-three SNPs showed the strongest association with MUO for the recessive, 12 for the codominant, and 32 for the dominant inheritance model (Supplementary Table S1).

### 2.3. Optimization of Genetic Risk Score and the Association of Optimized Genetic Risk Score with MUO and Related Parameters

The optimization process of genetic risk score (GRS) was performed based on SNPs that were shown to strengthen the association between GRS and MUO by logistic regression analysis, starting with the SNP with the strongest association (rs10838687: odds ratio (OR) = 1.92, 95% confidence intervals (95% CI): 1.26–2.93; *p* = 0.002) and moving step by step in decreasing order to the weakest (rs659366: OR = 1.03, 95% CI: 0.79–1.34; *p* = 0.844). During the process, 19 SNPs (for more details, see Supplementary Table S1) were selected, i.e., included in the present study.

**Figure 1:**
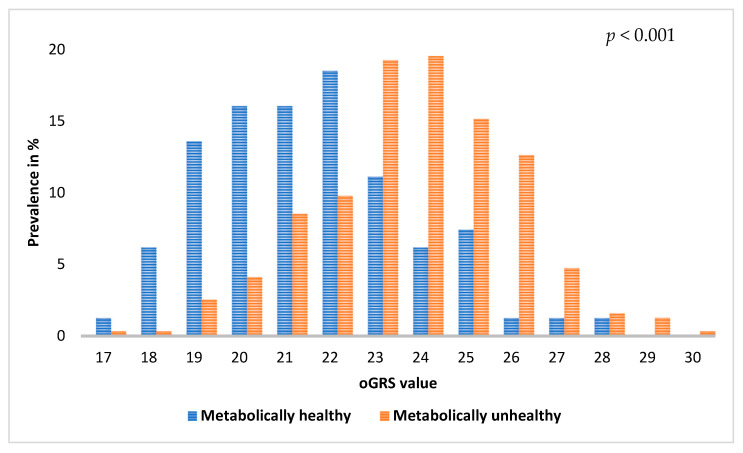
Comparison of the distribution of optimized genetic risk scores (oGRS) in metabolically healthy and unhealthy obese individuals. The genetic risk score was optimized for metabolically unhealthy obesity based on 19 single nucleotide polymorphisms (selected in the GRS optimization process).

The mean value of optimized GRS (oGRS) was 21.4 (95% CI: 20.9–21.9) in the MHO group and 23.7 (95% CI: 23.5–23.9) in the MUO group. The distribution of the oGRS showed a significant difference (*p* < 0.001) between the two groups, and significantly higher oGRS values were observed in the MUO group compared to the MHO group (Figure 1).

**Figure 2:**
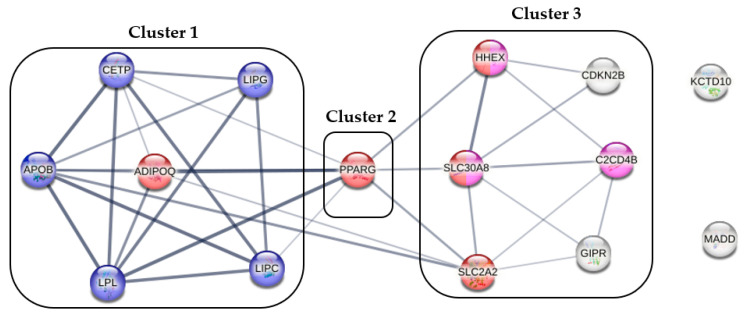
Gene–gene interaction and cluster analysis results based on genes containing SNPs selected during the genetic risk score optimization process. Note: genes associated with lipid metabolism are highlighted in blue, with diabetes in general in red, and with gestational diabetes in pink. The thickness of the lines connecting genes indicates the strength of the association between them.

The 19 identified SNPs are located in 15 genes, mainly in three main clusters (first cluster: *ADIPOQ*, *APOB*, *CETP*, *LIPC*, *LIPG* and *LPL*; second cluster: *PPARG*; and third cluster: *C2CD4B*, *CDKN2B*, *GIPR*, *HHEX*, *SLC2A2* and *SLC30A8*). The genes in the first cluster, with the exception of *ADIPOQ*, show a strong association with lipid metabolism, while genes in the second and third clusters are associated with type two and gestational diabetes. *KCTD10* and *MADD* genes could not be classified in either cluster (Figure 2).

None of the MUO-associated parameters tested (BMI, waist circumference (WC), SBP, DBP, fasting TAG, HDL-C, glucose, insulin, and homeostasis model assessment of insulin resistance (HOMA-IR)) showed any significant correlation with oGRS (after the test correction) (Supplementary Table S2).

### 2.4. The Discriminatory Power of MUO-Associated Genetic and Non-Genetic Risk Factors Based on ROC Curve Analyses

Age showed the highest discriminatory power (area under the receiver operating characteristic (ROC) curve (AUC)_age_ = 0.71, 95% CI: 0.63–0.78) among the conventional risk factors not considered among the MHO/MHO differential diagnostic criteria by Wildman et al. [26] and Meigs et al. [27] (sex, age, BMI, and education).

Among the physical and laboratory parameters used by them to define MUO (systolic and diastolic blood pressure, WC, fasting TAG, HDL-C, glucose, C-reactive protein (CRP), and HOMA-IR), TAG level showed the highest discriminatory power (AUC_TAG_ = 0.77, 95% CI: 0.72–0.82).

For oGRS, the power of discrimination was found to be AUC_oGRS_ = 0.77, 95% CI: 0.71–0.83. Based on a statistical comparison of the AUC curves, the discriminatory power of the oGRS was calculated by using the four most highly MUO-related SNPs (rs10838687 in MAP Kinase Activating Death Domain—*MADD*, rs693 in Apolipoprotein B—*APOB*, rs1111875 in Hematopoietically Expressed Homeobox—*HHEX* and rs2000813 in Endothelial lipase—*LIPG-*oGRS_4_) and was not significantly different from the oGRS calculated by involving the full panel of 19 SNPs included in the study (AUC_oGRS_ = 0.77 vs. AUC_oGRS4_ = 0.69, ΔAUC = 0.082, *p* = 0.014; Bonferroni corrected *p*-value < 0.0026).

**Figure 3:**
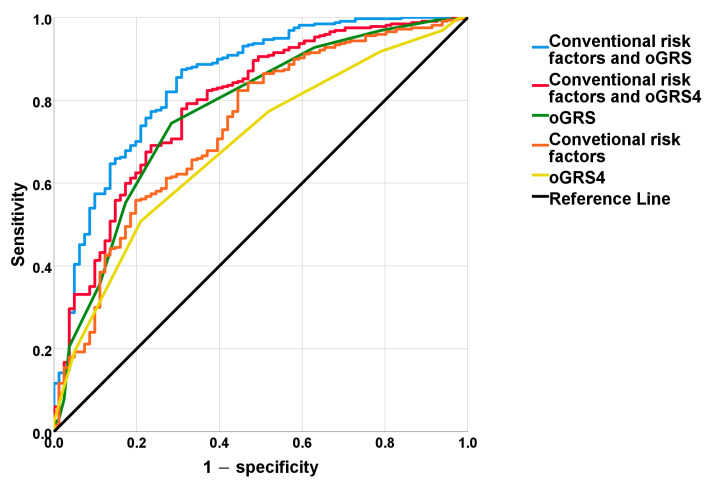
Results of receiver operating characteristic curve analysis for conventional risk factors (age, sex, education, and BMI—orange line) and genetic risk (based on the optimized genetic risk score (oGRS)—green line and oGRS_4_—yellow line) and the combination of conventional risk factors with oGRS (blue line) and the combination of conventional risk factors with oGRS_4_ (red line). The *y*-axis indicates sensitivity, and the *x*-axis indicates 1 − specificity. The black line represents a random predictive value of the model which is considered as a reference (area under the receiver operating characteristic (ROC) curve−AUC_ref._ = 0.500).

A combination of risk factors defined as conventional ones showed an overall discriminatory power of AUC_conv._ = 0.73 (95CI: 0.67–0.80). Conventional factors and oGRS_4_ together were as follows: AUC_conv.+oGRS4_ = 0.79 (95% CI: 0.74–0.85). Meanwhile, conventional and oGRS together were as follows: AUC_conv.+oGRS_ = 0.85 (95% CI: 0.80–0.90). The combination of both oGRS_4_ (AUC_conv._ = 0.73 vs. AUC_GRS4_ = 0.79, ΔAUC = 0.059; *p* = 0.007) and oGRS (AUC_conv._ = 0.73 vs. AUC_oGRS_ = 0.85, ΔAUC = 0.112; *p* <0.001) significantly improved the discrimination index compared to the one that only took into account the conventional risk factors (Figure 3).

**Figure 4:**
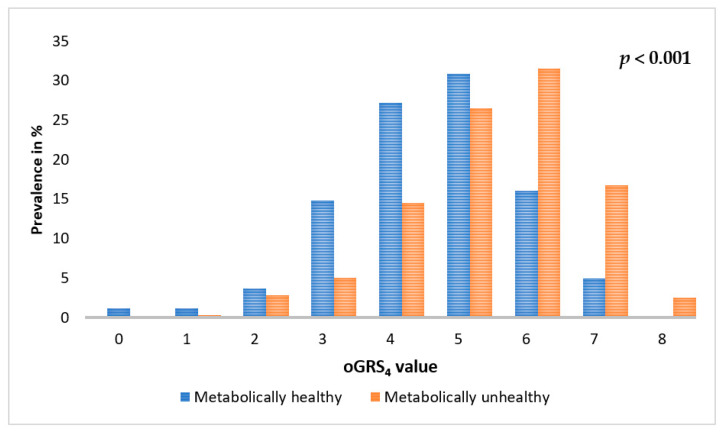
Comparison of the distribution of optimized genetic risk scores based on the four most strongly associated SNPs in metabolically healthy and unhealthy obese individuals.

The distribution of oGRS_4_ showed a significant difference between the MHO and MUO groups (MHO = 4.47, 95% CI: 4.17–4.77 vs. MUO = 5.38, 95% CI: 5.24–5.53; *p* < 0.001; Figure 4) and a significant correlation with MUO risk according to adjusted logistic regression analysis (OR = 1.76, 95% CI: 1.43–2.17; *p* = 8.77 × 10^−8^).

### 2.5. Association of oGRS with the Prevalence of Metabolically Unhealthy Obesity and with the Age of Individuals Affected

Based on oGRS, three genetic risk categories were formed (low, medium, and high), for which a significant trend was observed between higher genetic risk and a higher proportion of MUO individuals (prevalence of MUO in oGRS_low_: 53.8%, 95% CI: 43.6–63.7; oGRS_medium_: 85.2%, 95% CI: 80.3–89.3; and oGRS_high_: 95.6%, 95% CI: 88.7–98.7; *p* for trend <0.001).

**Figure 5:**
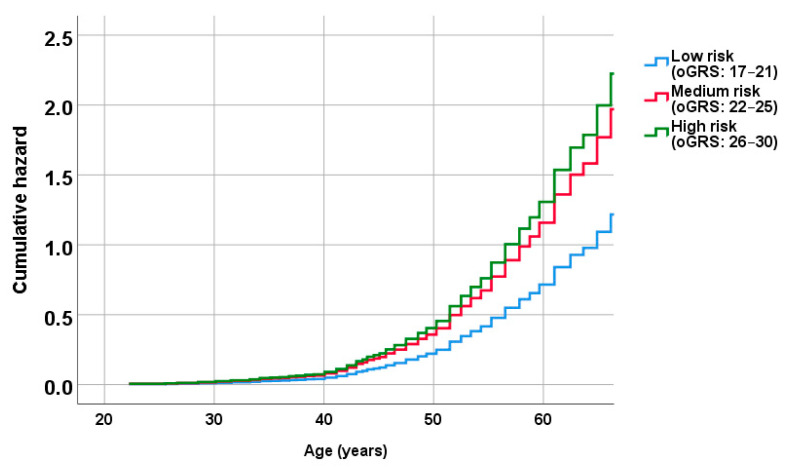
Cumulative risk of metabolically unhealthy obesity in relation to age in low (blue), medium (red) and high (green) genetic risk groups based on Cox regression proportional hazards model analysis.

In the absence of knowledge of the exact time of MUO onset, Cox regression analyses were performed using the individuals’ age at the time that the questionnaire was recorded. Cox regression analysis showed that oGRS as a continuous variable was significantly associated with an increased risk of developing MUO earlier (hazard ratio (HR) = 1.10, 95% CI: 1.05–1.15; *p* < 0.001). Among the genetic risk categories based on the oGRS, both in the medium (HR = 1.62, 95% CI: 1.18–2.21; *p* = 0.002) and the high (HR = 1.83, 95% CI: 1.26–2.65; *p* = 0.001) risk groups the risk of developing MHO at a younger age was significantly higher compared to the low-risk group (Figure 5).

### 2.6. Results of Trend and Multivariate Logistic Regression Analyses on the Association of oGRSs with the Metabolic Status

The results of the trend analyses show a significant increasing tendency in the average oGRS and oGRS_4_ values of the BMI subgroups in the metabolically unhealthy individuals (*p* < 0.001). Among the metabolically healthy individuals, only the average value of oGRS showed a significant result after *p*-value adjustment (Table 2).

The results of multivariate logistic regression analyses showed that oGRS, both separately (OR = 1.10, *p* = 0.00257) and in combination with BMI (OR = 1.07, *p* < 0.001), significantly increased the risk of metabolically unhealthy status in the total population (obese and non-obese together). oGRS_4_ separately did not show a significant association (OR = 1.15, *p* = 0.012) with metabolically unhealthy status, only in combination with BMI (OR = 1.01, *p* < 0.001) (Table 3).

## 3. Discussion

Our study is the first one to assess the genetic background variations that differentiate the MHO phenotype from the MUO one in a sample of Hungarian adults, based on genetic risk models involving SNPs associated with glucose homeostasis, lipid metabolism, and adiposity.

In the present study, we found a very high prevalence of MUO in the Hungarian obese sample population (79.6%). It is known that genetic factors, which affect metabolic pathways involved in adipogenesis, fat distribution, insulin signaling, and insulin resistance, can modulate the predisposition of developing obesity-related complications and lead to MUO [28]. In our oGRS models, we involved SNPs associated exactly with these CM traits to elucidate the links between the genetic background and the transition of MHO to MUO among individuals suffering from obesity.

The combined use of 19 SNPs (in 15 genes) that we examined showed a strong association of genetic factors with the MUO phenotype. In a GWAS-based study involving nearly 50,000 Koreans, it was found that polymorphisms in the *LPL*, *APOA5*, and *CETP* genes are associated with a higher risk of the metabolically unhealthy phenotype in the obese [29]. Furthermore, it was also shown that polymorphisms in the *CDKN2B* gene are also associated with the metabolically unhealthy phenotype even in normal-weight subjects. These results are in harmony with our findings.

Based on the gene–gene interaction analysis, the fifteen genes identified can be grouped into three clusters. The first cluster contains five genes related to lipid metabolism (*CETP*, *LIPG*, *APOB*, *LPL*, and *LIPC*) and one gene related to glucose metabolism (*ADIPOQ*). The *LPL* and *APIPOQ* genes of this cluster are associated with obesity induced by the consumption of high-fat foods [30] and show a strong association with the *PPARG* gene, which forms the second cluster. The results of an experiment in rodents suggest that the expression pattern of the *PPARG* gene is associated with high fat intake, adipocyte development, and insulin resistance [31]. The direct effects of the *ADIPOQ* and *PPARG* genes on plasma lipid profile and adiponectin concentration, as well as their interaction with diet, have been demonstrated in humans [32]. Dietary habits influence the association of six genes (*C2CD4B*, *CDKN2B*, *GIPR*, *HHEX*, *SLC2A2,* and *SLC30A8*) forming the third cluster with diabetes, adipogenesis, and cardiovascular risk [33,34,35,36].

Based on these, it is possible to conclude that the direct effects on bio-mechanisms of the gene clusters we identified are likely to be influenced by dietary factors as well. This assumption is further supported by the results of our multivariable logistic regression analyses, which demonstrate that genetic risk (defined as oGRS or oGRS_4_) in combination with an increase in BMI strongly contributes to the development of metabolically unhealthy status.

In the present study, we successfully identified a combination of four (rs10838687 in *MADD*, rs693 in *APOB*, rs1111875 in *HHEX,* and rs2000813 in *LIPG*) out of the 19 SNPs that significantly influence the risk of MUO developing. These sets of SNPs have been shown in previous studies to have significant effects on lipid and carbohydrate metabolisms. The rs10838687 in the *MADD* gene was found to be associated with a defect in the enzymatic conversion of proinsulin to insulin, resulting in increased fasting glucose levels, and with the development of T2D [37]. Based on our previous results, rs7944584 in linkage disequilibrium with rs10838687 is strongly associated with the early onset of insulin resistance in the Hungarian general and Roma populations [38]. The rs693 in the *APOB* gene increases cardiovascular risk [39,40] by raising the levels of APOB, TAG, TC, and LDL-C and reducing HDL-C [41] levels. The rs1111875 is located in the *HHEX* gene, which could be identified as a candidate gene for T2D using a genome-wide association approach. The association between *HHEX* and T2D has been reported in different ethnic groups [42]. The rs2000813 in the *LIPG* gene was found to be associated with lipid parameters and cardiovascular risk [43].

Based on our results, the CM markers most strongly associated with the risk of being MUO were BP, fasting glucose, HOMA-IR, and TAG, some of which are components of the metabolic syndrome (MetS). This is in line with a recent study, involving ten different cohorts from seven countries (*n* = 163,517 participants), which showed that BP, fasting glucose, and TAG were among the most frequent MetS components seen among MUO participants [44]. In this study, the most frequent MetS component among Finnish subjects suffering from obesity was elevated BP. In two other studies (i.e., one in Iran and one in Spain), dyslipidemia was found as the most frequent MetS component among obese individuals [45,46]. These parameters may be the most important indicators to predict the risk of metabolic deterioration to the MUO phenotype or the preservation of MHO status in the course of time.

A meta-analysis including eight longitudinal studies showed that MHO individuals are at increased risk for all-cause mortality in the long term (≥10 years), which indicates that MHO might be an intermediate stage of MUO [47] and people with MHO tend to develop metabolic dysregulation over time and have increased long-term CVD risk [48,49]. A pan-European cohort study (EPIC-CVD) showed that obese individuals without metabolic syndrome were at a higher risk of coronary heart disease than metabolically healthy individuals of normal weight (risk ratio (RR) = 1.28, 95% CI: 1.03–1.58, *p* = 0.001). Individuals with MHO have also a substantially higher risk for T2D than metabolically healthy individuals with normal weight (RR = 4.03, 95% CI: 2.66–6.09, *p* < 0.001).

The age difference of nearly 10 years between the MHO and MUO groups in our present study also supports the theory that the MHO is a dynamic condition and can transform into MUO over time [50,51,52], within 5.5 to 10.3 years of follow-up [51,52]. Our study model showed that a moderate to high genetic risk category was significantly associated with a lower mean age of participants with the MUO phenotype, strongly suggesting a link between genetic susceptibility to excessive fat adiposity and elevated CM disease risks in the early onset of obesity. Therefore, defining the variables that may predict the transition from metabolically healthy to unhealthy obesity in a specific population can help identify those who can benefit from it the most. Findings highlight the utility of potential interventions among MHO subjects with a higher susceptibility to MUO as a valid interim target, particularly in Hungary, one of the most obese countries in Europe [53].

In addition to the genetic background, other factors that may contribute to the transition of MHO to MUO have been studied in other populations. A prospective study conducted in a Spanish cohort (*n* = 3,052) found that any increase in BMI, waist size, or waist-to-hip ratio contributed to the transition from MHO to MUO, whereas adhering to a healthy dietary pattern, high levels of physical activity, and not smoking contributed to preventing this transition [50]. Future studies in Hungary should focus on refining and ascertaining specific factors that influence susceptibility to the transition to MUO, beyond our model.

All individuals suffering from obesity should aim for metabolic health and normal weight. Given our findings, it may be reasonable to consider genetic-based screening for obese or susceptible individuals to slow or even prevent the development of MUO. Early detection can help to avoid or at least mitigate the development of subsequent obesity-related complications (such as diabetes and cardiovascular disease). This approach is supported by the results of a study that examined the efficacy and safety of weight-loss drugs to prevent the development of T2D [54]. Participants were classified into three CM risk groups (low, medium, and high) based on their Cardiometabolic Disease Staging Score and it was found that although the preventive phentermine/topiramate medication reduced the risk of developing diabetes in all groups compared to the placebo (lifestyle intervention only) group, the reduction was significantly greater in the high-risk group compared to the medium and low groups. Therefore, targeting patients at high risk might improve the cost-to-benefit ratio of interventions.

It is important for professionals in the field of public health, healthcare research, and clinical practice, as well as patients, to acknowledge the complexity of factors and their interactions that contribute to the manifestation of obesity. This includes not only genetic (monogenic and polygenic), epigenetic, and developmental influences but also a multitude of interactions [55,56,57,58]. Currently, there is a renewed interest in defining models to explain the origins and development of obesity, leading to renewed debate. One proposed model, known as the Energy Balance Model (EBM), views overeating (consuming more calories than expended) as the primary cause of obesity. This model places emphasis on the role of unconscious signaling by the endocrine, metabolic, and nervous systems that control food intake [59], while highlighting the contribution of inexpensive, convenient, high in fat and sugar, “ultra-processed” (go through multiple processes) foods to the development of obesity. On the other hand, the Carbohydrate–Insulin Model (CIM) suggests that the hormonal response to highly processed carbohydrates plays a role in the partitioning of energy in the body, leading to increased deposition of fat in adipose tissue and reducing the calories available for the body’s metabolic needs [60]. This, in turn, can result in overeating to compensate for the sequestered calories. There have been efforts to reconcile these two models and create an integrated “push–pull” model of obesity pathogenesis [61]. Although the debate continues, public health action does not need to wait for a resolution, as both models identify major drivers of obesity and reflect the interactions of genes and the obesogenic environment.

The study has some limitations, which should be considered when interpreting our findings. Our results were not replicated and since the current study was performed in a European population, findings may not apply to non-European populations. Replication studies, including other populations, are necessary to confirm our findings and determine their applicability to ethnically diverse groups. Finally, although genetics do not change over time and it is possible to use this information prospectively, our study design remains cross-sectional, requiring future prospective studies to replicate and validate our results. Despite these stated limitations, we believe our study provides valuable information on the genetic characteristics of MUO and MHO phenotypes at a population level.

In conclusion, this study provides the first assessment on the genetic background of MHO and MUO phenotypes in a sample of Hungarian adults. Findings support the notion of early identification of individuals at high metabolic risk in populations suffering from obesity and show that in addition to environmental and lifestyle factors, one’s genetic background also has an important role in the development of MUO. Further, prospective study designs are warranted aiming at using genetic risk models not only to stratify the risk of impaired metabolic health among people suffering from obesity but also in normal-weight and overweight people. In summary, our study shows that obesity varies in its impact on metabolic health and renders unfavorable effects, offering a window of opportunity for early targeted public health interventions.

## 4. Materials and Methods

### 4.1. Sample Population and Relevant Parameters Defined

The sample was derived from a population-based disease-monitoring program, the General Practitioners’ Morbidity Sentinel Stations Program (GPMSSP) in Hungary [62]. Detailed methods of sampling and the data collection process are thoroughly described in the Hungarian Metabolic Syndrome Survey (HMSS) [63]. In brief, in the present study, 59 GPs from eight counties representing diverse socio-economic regions within Hungary were invited to participate. Medical and socio-demographic characteristics were recorded and physical examinations (weight, height, waist circumference, and blood pressure measurements) were carried out. Blood samples were collected for laboratory tests (including routine diagnostic tests for fasting glucose, insulin, C-reactive protein, HDL-cholesterol, and triacylglycerols) and DNA isolation. HOMA-IR was calculated according to the following formula: fasting insulin (microU/L) x fasting glucose (nM)/22.5. Medications for hypertension, diabetes, and lipid disturbances were also recorded.

**Figure 6:**
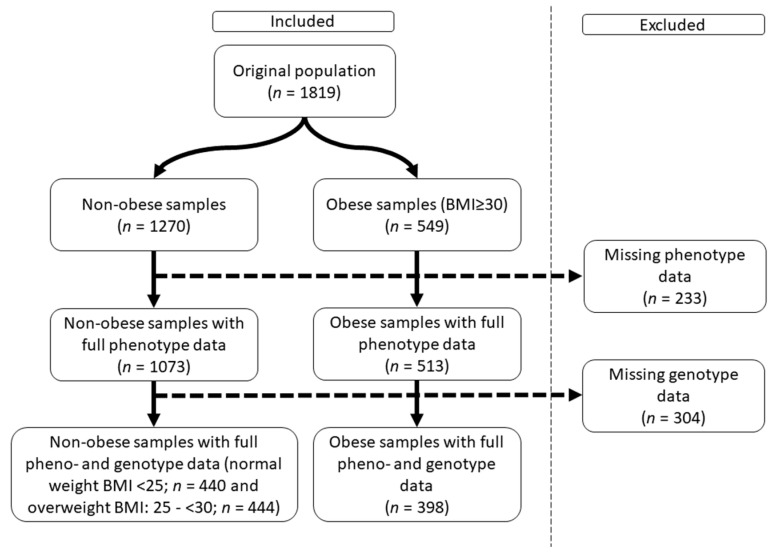
Flow chart of the study sample selection.

Initially, data from 1819 participants representing 91% of invited individuals were collected. In the present study, those with complete geno-/phenotype data (*n* = 1282) were included. The total population was divided into three subgroups based on BMI: normal weight (BMI < 25; *n* = 440), overweight (BMI: 25–< 30; *n* = 444), and obese (BMI ≥ 30; *n* = 398) (Figure 6).

### 4.2. Defining Metabolically Healthy and Unhealthy Obesity

There is no universally accepted definition for the MHO and MUO phenotypes; therefore, MHO and MUO subjects in this analysis were identified by using a combination of classifying criteria established by Wildman et al. [26] and Meigs et al. [27] (Table 4). This was achieved by considering (a) the robustness of these criteria and (b) the availability of variables in our database.

### 4.3. DNA Isolation, SNP Selection and Genotyping

DNA was isolated using a MagNA Pure LC system (Roche Diagnostics, Basel, Switzerland) with a MagNA Pure LC DNA Isolation Kit—Large Volume according to prespecified instructions of the manufacturer. Extracted DNA was eluted in 200 μL MagNA Pure LC DNA Isolation Kit—Large Volume elution buffer.

SNPs strongly associated with obesity, lipid metabolism, and glucose homeostasis were identified by screening PubMed, HuGE Navigator, and Ensembl databases. As a result, a total of 67 SNPs (in 44 genes) were considered (Supplementary Table S1), of which (a) 23 were most strongly associated with obesity [64], (b) 22 with lipid metabolism [65], and (c) 22 with glucose homeostasis [66]. Concerning the effects of SNPs regarding the metabolic traits, overlaps are possible.

Genotyping was performed using the MassARRAY platform (Sequenom Inc., San Diego, CA, USA) with iPLEX Gold chemistry by the Mutation Analysis Core Facility at the Karolinska University Hospital, Sweden. Validation, concordance analysis, and quality control were conducted by the Facility according to their protocols.

### 4.4. Identification and Coding of the Genetic Model Best Associated with HOMA-IR by SNPs

For each SNP, three widely used genetic inheritance models (i.e., codominant, dominant, and recessive) were examined to determine which model had the strongest association with MUO as a binary outcome (i.e., MUO vs. MHO). Multivariable logistic analysis (controlled for age, sex, and education) was conducted to test each SNP’s association with MUO. Cox and Snell R^2^ (the higher the better) and *p*-values (the lower the better) guided the selection process of the best-fitted heritability model [67]. We considered the most suitable heritability model associated with MUO for each SNP used in the optimized genetic risk score (oGRS).

Coding for each SNP was based on the following genetic model of inheritance criteria:

a) Codominant genetic model: homozygote genotype with risk allele was labelled as 2, whereas heterozygote gene labelled as 1 and 0 was coded for no risk allele.
b) Dominant genetic model: 2 was coded for the presence of one or two risk alleles and 0 was coded for the absence of a risk allele.
c) Recessive genetic model: 2 was counted for the presence of two risk alleles, while 0 was counted for the homozygote gene with the absence of a risk allele and for the heterozygote gene.

### 4.5. Calculation and Optimization of the Genetic Risk Score

The oGRS was calculated using the following equation:

$$oGRS=\sum_{i=1}^{I}G_{i}$$

where *G_i_* is the risk score according to the chosen heritability model (see the previous subsection). The genetic risk model optimization procedure selected SNPs with the strongest association with MUO (as a binary outcome variable). GRS optimization was performed using multivariable logistic analyses. The SNPs were tested in ascending order of *p*-value and each SNP was inserted into the model successively, starting from the SNP with the strongest association (lowest *p*-value), and the association between oGRS and MUO was examined after each succession. SNPs were selected and used for the final optimized GRS only if they increased the strength of association of oGRS with MUO. SNPs that did not affect or weakened the association were excluded from further analyses. Based on the oGRS, individuals were classified into three genetic risk groups based on tertiles. Individuals in the lowest tertile were assigned to the low-risk group, the individuals in the second tertile were assigned to the moderate-risk group, and the individuals in the third tertile were assigned to the high-risk group.

### 4.6. Statistical Analysis

The statistical procedures used to develop the genetic risk were tested and developed on the obesity sample population (*n* = 398), while its interaction with and separately from BMI was tested on the total population (obese and non-obese subpopulation, *n* = 1282). Chi-square (χ^2^) was used to test the Hardy–Weinberg equilibrium (HWE) of genotyped SNPs and compare differences between non-quantitative variables within the study population. The Shapiro–Wilk test was used to examine whether the quantitative variables are normally distributed, and, if necessary, Templeton’s two-step method [68] was considered to transform the non-normal variables into normal ones. The Mann–Whitney U test was used to assess the distribution of non-normally distributed data between the study groups. Multivariable logistic analyses were used to determine the association between individual SNPs, the aggregate of them (oGRS), and MUO. Cox regression analysis was used to examine the association of oGRS with age at the onset of MUO. In these analyses, the age of the individual at the time the questionnaire was collected was used as the outcome variable. All regression analyses were carried out under an adjusted model. The online software Search Tool for the Retrieval of Interacting genes (STRING-version 11.5; https://string-db.org/; accessed on 10 January 2023) was used for interaction and cluster analysis and visualization of genes and proteins [69]. A minimum interaction score of 0.400 was used and Markov Cluster Algorithm was applied for determining clusters.

The association of risk groups (low, medium, and high) based on oGRS scores with age-at-event (age at identifying MUO in the survey) was examined using multivariable logistic regression (i.e., adjusted for age, sex, BMI, and education). A statistically significant trend between the proportion of individuals with MUO and oGRS risk categories was tested with the Jonckheere-Terpstra test [70]. Receiver operating characteristic (ROC) analysis was employed to evaluate the discriminatory ability of the oGRS and the area under the curve (AUC) was used as an indicator of diagnostic accuracy. In addition, the minimum number of SNPs for which the discrimination accuracy is not significantly different compared to the oGRS was also determined on the bases of the ROC curves’ analyses and the effect of them was also examined.

Multivariate logistic regression analysis was used to investigate the association between the genetic risk (defined by oGRS or oGRS_4_) and its interaction with BMI and the risk of developing metabolically unhealthy conditions for the total study population. In the statistical analysis of interaction variables, adjustments were made for age, sex, education, BMI, and oGRSs.

Statistical tests were carried out using IBM SPSS version 26 statistics for Windows (Armonk, NY, United States). Bonferroni-corrected *p*-value was established for the case where several dependent or independent statistical tests were performed simultaneously on a single data set.

## Figures and Tables

**Table 1:** Characteristics of obese participants by metabolically healthy and unhealthy status.

		MHO (*n* = 81)	MUO (*n* = 317)	*p*-Value
		Average (95% CI)		
Age (years)		45.5 (42.7–48.2)	54.4 (53.2–55.5)	<0.001
BMI (kg/m^2^)		33.1 (32.4–33.8)	34.5 (34.1–35.0)	0.001
Waist circumference (cm)		106.9 (104.5–109.2)	112.9 (111.5–114.2)	<0.001
Systolic blood pressure (mmHg)		127.3 (123.9–130.7)	141.0 (1392–142.8)	<0.001
Diastolic blood pressure (mmHg)		80.7 (78.9–82.5)	85.0 (84.0–86.1)	<0.001
Fasting TAG level (mM)		1.20 (1.09–1.30)	2.21 (2.02–2.39)	<0.001
Fasting HDL-C level (mM)		1.45 (1.45–1.52)	1.20 (1.17–1.24)	<0.001
Fasting glucose level (mM)		4.33 (4.16–4.51)	5.56 (5.33–5.79)	<0.001
Fasting insulin level (mM)		186.57 (165.36–207.79)	278.59 (256.62–300.57)	<0.001
HOMA-IR		5.29 (4.59–5.98)	10.98 (9.57–12.40)	<0.001
CRP concentration (nM)		28.86 (23.66–34.06)	45.52 (40.75–50.30)	<0.001
		**Prevalence in % (95% CI)**		***p*-Value**
Women		46.9 (36.3–57.7)	47.6 (42.2–53.1)	0.908
Anti-hypertensive treatment		0.0	75.4 (70.4–79.9)	<0.001
Anti-diabetic treatment		0.0	16.1 (12.4–20.4)	<0.001
Lipid-lowering treatment		6.2 (2.4–13.0)	31.5 (26.6–36.8)	<0.001
Education	Less than primary or primary	28.4 (19.5–38.8)	28.7 (23.9–33.9)	0.846
	High or vocational school	63.0 (52.1–72.9)	60.6 (55.1–65.8)	
	College or university	8.6 (3.9–16.2)	10.7 (7.7–14.5)	

Note: 95% CI: 95% confidence intervals; BMI: body mass index; MUO: metabolically unhealthy obese; MHO: metabolically healthy obese; TAG: triacylglycerols; HDL-C: high-density lipoprotein cholesterol; HOMA-IR: homeostasis model assessment of insulin resistance; CRP: C-reactive protein.

**Table 2:** Results of trend analyses carried out on optimized genetic risk score (oGRS) and oGRS_4_ values changing by subgroups created on the basis of BMI values.

	Normal Weight BMI < 25	Overweight BMI: 25– < 30	Obese BMI ≥ 30	*p* for Trend
	Average oGRS Value (95% CI)			
Metabolically healthy	23.18 (22.94–23.42)	23.27 (22.96–23.58)	21.44 (20.95–21.94)	<0.001 **
Metabolically unhealthy	22.65 (21.88–23.41)	23.05 (22.72–23.39)	23.72 (23.48–23.95)	<0.001 **
	**Average oGRS_4_ Value (95% CI)**			***p* for Trend**
Metabolically healthy	5.07 (4.94–5.20)	5.10 (4.93–5.28)	4.47 (4.17–4.77)	0.033 *
Metabolically unhealthy	4.92 (4.52–5.32)	5.04 (4.85–5.23)	5.38 (5.24–5.53)	<0.001 **

* *p* < 0.05; ** significance threshold determined after Bonferroni test correction (*p* < 0.0026); 95% CI: 95% confidence intervals.

**Table 3:** Results of multivariate logistic regression analyses of optimized genetic risk score (oGRS) and oGRS_4_ separately and in combination with BMI (as an interaction variable) on the risk of metabolically unhealthy status.

	OR (95% CI)	*p*-value
oGRS	1.10 (1.03–1.17)	0.00257 **
oGRS *×* BMI	1.07 (1.05–1.08)	<0.001 **
oGRS_4_	1.15 (1.03–1.28)	0.012 *
oGRS_4_ *×* BMI	1.01 (1.00–1.01)	0.001 **

95% CI: 95% confidence intervals; *x*: interacting variables; * *p* < 0.05; ** significance threshold determined after Bonferroni test correction: *p* < 0.0026.

**Table 4:** Criteria for definition of metabolically healthy obese (MHO) and metabolically unhealthy obese (MUO) phenotypes.

	Wildman et al. [26]	Meigs et al. [27]
BP ≥ 130/85 mmHg	X	X
Use of blood pressure medication	X	X
Fasting TAG level ≥ 1.7 mM (≥150 mg/dL)	X	X
HDL cholesterol 1 mM (≥40 mg/dL) in men or 1.3 mM (50 mg/dL) in women	X	X
Lipid-lowering medication	X	
Fasting glucose level 5.6 mM (≥100 mg/dL)	X	X
Use of medications for diabetes	X	
Waist circumference > 102 cm in men or > 88 cm in women		X
HOMA-IR ≥ 90% among those with BMI ≥ 30 kg/m^2^	X	
C-reactive protein ≥ 90% among those with BMI ≥ 30 kg/m^2^	X	
Criteria for MUO	>1	>2

BP: blood pressure; TAG: triacylglycerols; HDL: high-density lipoprotein; HOMA-IR: homeostasis model assessment of insulin resistance; BMI: body mass index.

## Data Availability

Data are available on request due to privacy or ethical concerns.

## Supplementary Materials

The following supporting information can be downloaded at https://www.mdpi.com/article/10.3390/ijms24065209/s1.

## References

[1] Swinburn B.A., Kraak V.I., Allender S., Atkins V.J., Baker P.I., Bogard J.R., Brinsden H., Calvillo A., De Schutter O., Devarajan R. (2019). The Global Syndemic of Obesity, Undernutrition, and Climate Change: The Lancet Commission report. Lancet.

[2] Wang L., Southerland J., Wang K., Bailey B.A., Alamian A., Stevens M.A., Wang Y. (2017). Ethnic Differences in Risk Factors for Obesity among Adults in California, the United States. J. Obes..

[3] Shai I., Jiang R., Manson J.E., Stampfer M.J., Willett W.C., Colditz G.A., Hu F.B. (2006). Ethnicity, obesity, and risk of type 2 diabetes in women: A 20-year follow-up study. Diabetes Care.

[4] Wen C.P., David Cheng T.Y., Tsai S.P., Chan H.T., Hsu H.L., Hsu C.C., Eriksen M.P. (2009). Are Asians at greater mortality risks for being overweight than Caucasians? Redefining obesity for Asians. Public Health Nutr..

[5] Pischon T., Boeing H., Hoffmann K., Bergmann M., Schulze M.B., Overvad K., van der Schouw Y.T., Spencer E., Moons K.G., Tjonneland A. (2008). General and abdominal adiposity and risk of death in Europe. N. Engl. J. Med..

[6] Malik V.S., Willett W.C., Hu F.B. (2013). Global obesity: Trends, risk factors and policy implications. Nat. Rev. Endocrinol..

[7] Stefan N., Haring H.U., Hu F.B., Schulze M.B. (2013). Metabolically healthy obesity: Epidemiology, mechanisms, and clinical implications. Lancet Diabetes Endocrinol..

[8] Finucane M.M., Stevens G.A., Cowan M.J., Danaei G., Lin J.K., Paciorek C.J., Singh G.M., Gutierrez H.R., Lu Y., Bahalim A.N. (2011). National, regional, and global trends in body-mass index since 1980: Systematic analysis of health examination surveys and epidemiological studies with 960 country-years and 9.1 million participants. Lancet.

[9] Bray G.A., Kim K.K., Wilding J.P.H., World Obesity F. (2017). Obesity: A chronic relapsing progressive disease process. A position statement of the World Obesity Federation. Obes. Rev..

[10] Sims E.A. (2001). Are there persons who are obese, but metabolically healthy?. Metabolism.

[11] Tsatsoulis A., Paschou S.A. (2020). Metabolically Healthy Obesity: Criteria, Epidemiology, Controversies, and Consequences. Curr. Obes. Rep..

[12] Bluher M. (2020). Metabolically Healthy Obesity. Endocr. Rev..

[13] Eckel N., Meidtner K., Kalle-Uhlmann T., Stefan N., Schulze M.B. (2016). Metabolically healthy obesity and cardiovascular events: A systematic review and meta-analysis. Eur. J. Prev. Cardiol.

[14] Stefan N., Haring H.U., Schulze M.B. (2018). Metabolically healthy obesity: The low-hanging fruit in obesity treatment?. Lancet Diabetes Endocrinol..

[15] Zembic A., Eckel N., Stefan N., Baudry J., Schulze M.B. (2021). An Empirically Derived Definition of Metabolically Healthy Obesity Based on Risk of Cardiovascular and Total Mortality. JAMA Netw. Open.

[16] Rey-Lopez J.P., de Rezende L.F., Pastor-Valero M., Tess B.H. (2014). The prevalence of metabolically healthy obesity: A systematic review and critical evaluation of the definitions used. Obes. Rev..

[17] Albuquerque D., Nobrega C., Manco L., Padez C. (2017). The contribution of genetics and environment to obesity. Br. Med. Bull..

[18] Herrera B.M., Lindgren C.M. (2010). The genetics of obesity. Curr. Diab Rep..

[19] Goodarzi M.O. (2018). Genetics of obesity: What genetic association studies have taught us about the biology of obesity and its complications. Lancet Diabetes Endocrinol..

[20] Navarro E., Funtikova A.N., Fito M., Schroder H. (2015). Can metabolically healthy obesity be explained by diet, genetics, and inflammation?. Mol. Nutr. Food Res..

[21] Berezina A., Belyaeva O., Berkovich O., Baranova E., Karonova T., Bazhenova E., Brovin D., Grineva E., Shlyakhto E. (2015). Prevalence, Risk Factors, and Genetic Traits in Metabolically Healthy and Unhealthy Obese Individuals. Biomed. Res. Int..

[22] Sedaghati-Khayat B., Barzin M., Akbarzadeh M., Guity K., Fallah M.S., Pourhassan H., Azizi F., Daneshpour M.S. (2020). Lack of association between FTO gene variations and metabolic healthy obese (MHO) phenotype: Tehran Cardio-metabolic Genetic Study (TCGS). Eat Weight Disord..

[23] Dixon J.B. (2010). The effect of obesity on health outcomes. Mol. Cell Endocrinol..

[24] Zhang Z.Y., Wang M.W. (2012). Obesity, a health burden of a global nature. Acta Pharmacol. Sin..

[25] Okunogbe A., Nugent R., Spencer G., Ralston J., Wilding J. (2021). Economic impacts of overweight and obesity: Current and future estimates for eight countries. BMJ Glob. Health.

[26] Wildman R.P., Muntner P., Reynolds K., McGinn A.P., Rajpathak S., Wylie-Rosett J., Sowers M.R. (2008). The obese without cardiometabolic risk factor clustering and the normal weight with cardiometabolic risk factor clustering: Prevalence and correlates of 2 phenotypes among the US population (NHANES 1999–2004). Arch Intern. Med..

[27] Meigs J.B., Wilson P.W., Fox C.S., Vasan R.S., Nathan D.M., Sullivan L.M., D’Agostino R.B. (2006). Body mass index, metabolic syndrome, and risk of type 2 diabetes or cardiovascular disease. J. Clin. Endocrinol. Metab..

[28] Huang L.O., Loos R.J.F., Kilpelainen T.O. (2018). Evidence of genetic predisposition for metabolically healthy obesity and metabolically obese normal weight. Physiol. Genom..

[29] Park J.M., Park D.H., Song Y., Kim J.O., Choi J.E., Kwon Y.J., Kim S.J., Lee J.W., Hong K.W. (2021). Understanding the genetic architecture of the metabolically unhealthy normal weight and metabolically healthy obese phenotypes in a Korean population. Sci. Rep..

[30] Walton R.G., Zhu B.B., Unal R., Spencer M., Sunkara M., Morris A.J., Charnigo R., Katz W.S., Daugherty A., Howatt D.A. (2015). Increasing Adipocyte Lipoprotein Lipase Improves Glucose Metabolism in High Fat Diet-induced Obesity. J. Biol. Chem..

[31] VidalPuig A., JimenezLinan M., Lowell B.B., Hamann A., Hu E., Spiegelman B., Flier J.S., Moller D.E. (1996). Regulation of PPAR gamma gene expression by nutrition and obesity in rodents. J. Clin. Investig..

[32] AlSaleh A., Sanders T.A.B., O’Dell S.D. (2012). Postgraduate Symposium Effect of interaction between PPARG, PPARA and ADIPOQ gene variants and dietary fatty acids on plasma lipid profile and adiponectin concentration in a large intervention study. Proc. Nutr. Soc..

[33] Evseeva M.N., Dyikanov D.T., Karagyaur M.N., Prikazchikova T.A., Sheptulina A.F., Balashova M.S., Zatsepin T.S., Rubtsov Y.P., Kulebyakin K.Y. (2021). Hematopoietically-expressed homeobox protein HHEX regulates adipogenesis in preadipocytes. Biochimie.

[34] Narasimhan A., Chinnaiyan M., Karundevi B. (2015). Ferulic acid regulates hepatic GLUT2 gene expression in high fat and fructose-induced type-2 diabetic adult male rat. Eur. J. Pharmacol..

[35] Mehramiz M., Ghasemi F., Esmaily H., Tayefi M., Hassanian S.M., Sadeghzade M., Sadabadi F., Moohebati M., Azarpazhooh M.R., Parizadeh S.M.R. (2018). Interaction between a variant of CDKN2A/B-gene with lifestyle factors in determining dyslipidemia and estimated cardiovascular risk: A step toward personalized nutrition. Clin. Nutr..

[36] Qi Q.B., Bray G.A., Hu F.B., Sacks F.M., Qi L. (2012). Weight-loss diets modify glucose-dependent insulinotropic polypeptide receptor rs2287019 genotype effects on changes in body weight, fasting glucose, and insulin resistance: The Preventing Overweight Using Novel Dietary Strategies trial. Am. J. Clin. Nutr..

[37] Strawbridge R.J., Dupuis J., Prokopenko I., Barker A., Ahlqvist E., Rybin D., Petrie J.R., Travers M.E., Bouatia-Naji N., Dimas A.S. (2011). Genome-wide association identifies nine common variants associated with fasting proinsulin levels and provides new insights into the pathophysiology of type 2 diabetes. Diabetes.

[38] Piko P., Werissa N.A., Fiatal S., Sandor J., Adany R. (2020). Impact of Genetic Factors on the Age of Onset for Type 2 Diabetes Mellitus in Addition to the Conventional Risk Factors. J. Pers. Med..

[39] Park M.H., Kim N., Lee J.Y., Park H.Y. (2011). Genetic loci associated with lipid concentrations and cardiovascular risk factors in the Korean population. J. Med. Genet..

[40] Nikolajevic Starcevic J., Santl Letonja M., Praznikar Z.J., Makuc J., Vujkovac A.C., Petrovic D. (2014). Polymorphisms XbaI (rs693) and EcoRI (rs1042031) of the ApoB gene are associated with carotid plaques but not with carotid intima-media thickness in patients with diabetes mellitus type 2. Vasa.

[41] Niu C., Luo Z., Yu L., Yang Y., Chen Y., Luo X., Lai F., Song Y. (2017). Associations of the APOB rs693 and rs17240441 polymorphisms with plasma APOB and lipid levels: A meta-analysis. Lipids Health Dis..

[42] Wang Y., Qiao W., Zhao X., Tao M. (2011). Quantitative assessment of the influence of hematopoietically expressed homeobox variant (rs1111875) on type 2 diabetes risk. Mol. Genet. Metab..

[43] Abudureyimu S., Abulaiti P., Li H., Xing Z., Liu S., Li W., Gao Y. (2021). Roles of endothelial lipase gene related single nucleotide polymorphisms in patients with coronary artery disease. Gene.

[44] Van Vliet-Ostaptchouk J.V., Nuotio M.L., Slagter S.N., Doiron D., Fischer K., Foco L., Gaye A., Gogele M., Heier M., Hiekkalinna T. (2014). The prevalence of metabolic syndrome and metabolically healthy obesity in Europe: A collaborative analysis of ten large cohort studies. BMC Endocr. Disord..

[45] Tabatabaei-Malazy O., Saeedi Moghaddam S., Masinaei M., Rezaei N., Mohammadi Fateh S., Dilmaghani-Marand A., Abdolhamidi E., Razi F., Khashayar P., Mahdavihezaveh A. (2022). Association between being metabolically healthy/unhealthy and metabolic syndrome in Iranian adults. PLoS ONE.

[46] Gutierrez-Repiso C., Linares-Pineda T.M., Gonzalez-Jimenez A., Aguilar-Lineros F., Valdes S., Soriguer F., Rojo-Martinez G., Tinahones F.J., Morcillo S. (2021). Epigenetic Biomarkers of Transition from Metabolically Healthy Obesity to Metabolically Unhealthy Obesity Phenotype: A Prospective Study. Int. J. Mol. Sci..

[47] Kramer C.K., Zinman B., Retnakaran R. (2013). Are metabolically healthy overweight and obesity benign conditions?: A systematic review and meta-analysis. Ann. Intern. Med..

[48] Mongraw-Chaffin M., Foster M.C., Anderson C.A.M., Burke G.L., Haq N., Kalyani R.R., Ouyang P., Sibley C.T., Tracy R., Woodward M. (2018). Metabolically Healthy Obesity, Transition to Metabolic Syndrome, and Cardiovascular Risk. J. Am. Coll. Cardiol..

[49] Deedwania P., Lavie C.J. (2018). Dangers and Long-Term Outcomes in Metabolically Healthy Obesity: The Impact of the Missing Fitness Component. J. Am. Coll. Cardiol..

[50] Schroder H., Ramos R., Baena-Diez J.M., Mendez M.A., Canal D.J., Fito M., Sala J., Elosua R. (2014). Determinants of the transition from a cardiometabolic normal to abnormal overweight/obese phenotype in a Spanish population. Eur. J. Nutr..

[51] Soriguer F., Gutierrez-Repiso C., Rubio-Martin E., Garcia-Fuentes E., Almaraz M.C., Colomo N., Esteva de Antonio I., de Adana M.S., Chaves F.J., Morcillo S. (2013). Metabolically healthy but obese, a matter of time? Findings from the prospective Pizarra study. J. Clin. Endocrinol. Metab..

[52] Appleton S.L., Seaborn C.J., Visvanathan R., Hill C.L., Gill T.K., Taylor A.W., Adams R.J., North West Adelaide Health Study T. (2013). Diabetes and cardiovascular disease outcomes in the metabolically healthy obese phenotype: A cohort study. Diabetes Care.

[53] WHO (2022). WHO European Regional Obesity Report 2022.

[54] Guo F., Garvey W.T. (2017). Cardiometabolic Disease Staging Predicts Effectiveness of Weight-Loss Therapy to Prevent Type 2 Diabetes: Pooled Results From Phase III Clinical Trials Assessing Phentermine/Topiramate Extended Release. Diabetes Care.

[55] Walley A.J., Asher J.E., Froguel P. (2009). The genetic contribution to non-syndromic human obesity. Nat. Rev. Genet..

[56] Choquet H., Meyre D. (2011). Genetics of Obesity: What have we Learned?. Curr. Genom..

[57] Herrera B.M., Keildson S., Lindgren C.M. (2011). Genetics and epigenetics of obesity. Maturitas.

[58] Thompson A.L. (2012). Developmental origins of obesity: Early feeding environments, infant growth, and the intestinal microbiome. Am. J. Hum. Biol..

[59] Hall K.D., Farooqi I.S., Friedman J.M., Klein S., Loos R.J.F., Mangelsdorf D.J., O’Rahilly S., Ravussin E., Redman L.M., Ryan D.H. (2022). The energy balance model of obesity: Beyond calories in, calories out. Am. J. Clin. Nutr..

[60] Ludwig D.S., Aronne L.J., Astrup A., de Cabo R., Cantley L.C., Friedman M.I., Heymsfield S.B., Johnson J.D., King J.C., Krauss R.M. (2021). The carbohydrate-insulin model: A physiological perspective on the obesity pandemic. Am. J. Clin. Nutr..

[61] Ludwig D.S., Sorensen T.I.A. (2022). An integrated model of obesity pathogenesis that revisits causal direction. Nat. Rev. Endocrinol..

[62] Szeles G., Voko Z., Jenei T., Kardos L., Pocsai Z., Bajtay A., Papp E., Pasti G., Kosa Z., Molnar I. (2005). A preliminary evaluation of a health monitoring programme in Hungary. Eur. J. Public Health.

[63] Szigethy E., Szeles G., Horvath A., Hidvegi T., Jermendy G., Paragh G., Blasko G., Adany R., Voko Z. (2012). Epidemiology of the metabolic syndrome in Hungary. Public Health.

[64] Nagy K., Fiatal S., Sandor J., Adany R. (2017). Distinct Penetrance of Obesity-Associated Susceptibility Alleles in the Hungarian General and Roma Populations. Obes. Facts.

[65] Piko P., Fiatal S., Kosa Z., Sandor J., Adany R. (2017). Genetic factors exist behind the high prevalence of reduced high-density lipoprotein cholesterol levels in the Roma population. Atherosclerosis.

[66] Werissa N.A., Piko P., Fiatal S., Kosa Z., Sandor J., Adany R. (2019). SNP-Based Genetic Risk Score Modeling Suggests No Increased Genetic Susceptibility of the Roma Population to Type 2 Diabetes Mellitus. Genes.

[67] Salanti G., Southam L., Altshuler D., Ardlie K., Barroso I., Boehnke M., Cornelis M.C., Frayling T.M., Grallert H., Grarup N. (2009). Underlying genetic models of inheritance in established type 2 diabetes associations. Am. J. Epidemiol..

[68] Templeton G.F., Pope M.B., Burney L.L. (2021). The Usefulness of the Two-Step Normality Transformation in Retesting Existing Theories: Evidence on the Productivity Paradox. Data Base Adv. Inf. Syst..

[69] Szklarczyk D., Gable A.L., Lyon D., Junge A., Wyder S., Huerta-Cepas J., Simonovic M., Doncheva N.T., Morris J.H., Bork P. (2019). STRING v11: Protein-protein association networks with increased coverage, supporting functional discovery in genome-wide experimental datasets. Nucleic Acids Res..

[70] Jonckheere A.R. (1954). A Distribution-free k-sample test against ordered alternatives. Biometrika.

