# Supplementary material for: Genetic Background of Metabolically Healthy and Unhealthy Obesity Phenotypes in Hungarian Adult Sample Population
Source: Int J Mol Sci. 2023 Mar 8;24(6):5209. doi: 10.3390/ijms24065209 (PMC10049500; doi:10.3390/ijms24065209)
Supplement: Supplementary file 1 [file ijms-24-05209-s001.zip › Supplementary Table 1.pdf]

International Journal of
Molecular Sciences

MDPI

Supplementary Table S1. A summary table of single nucleotide polymorphisms (SNPs) investigated (included or excluded), their risk alleles in the genes affected, their best-fitted inheritance models for metabolically unhealthy obesity (MUO), the typical biochemical traits affected, the genetic risk score (GRS) optimization process Cox & Snell R Squares- and *p*-values.

No.	SNP	Gene	Risk allele	Best-fitted models of inheritance	Trait	Cox & Snell R Square	*p*-value	Optimized GRS
1	rs10838687	*MADD*	T	codominant	Glucose homeostasis	0.125	2.43E-03	Included
2	rs693	*APOB*	G	codominant	Lipid metabolism	0.144	3.90E-05	Included
3	rs1111875	*HHEX*	C	codominant	Glucose homeostasis	0.163	9.29E-07	Included
4	rs2000813	*LIPG*	C	codominant	Lipid metabolism	0.175	8.77E-08	Included
5	rs1801282	*PPARG*	C	recessive	Obesity	0.194	2.46E-09	Included
6	rs11558471	*SLC30A8*	G	codominant	Glucose homeostasis	0.202	5.29E-10	Included
7	rs2241766	*ADIPOQ*	T	dominant	Obesity	0.209	1.45E-10	Included
8	rs328	*LPL*	C	dominant	Lipid metabolism	0.212	8.00E-11	Included
9	rs3846662	*HMGCR*	A	dominant	Lipid metabolism	0.210	9.70E-11	Excluded
10	rs1532624	*CETP*	C	recessive	Lipid metabolism	0.221	2.16E-11	Included
11	rs10830963	*MTNR1B*	G	recessive	Glucose homeostasis	0.220	3.11E-11	Excluded
12	rs2144300	*GALNT2*	C	dominant	Lipid metabolism	0.216	3.57E-11	Excluded
13	rs4846914	*GALNT2*	G	dominant	Lipid metabolism	0.216	3.57E-11	Excluded
14	rs708272	*CETP*	G	recessive	Lipid metabolism	0.197	2.89E-09	Excluded
15	rs3736594	*MRPL33*	A	codominant	Glucose homeostasis	0.220	2.44E-11	Excluded
16	rs174550	*FADS1*	T	dominant	Glucose homeostasis	0.206	3.16E-10	Excluded
17	rs1387153	*MTNR1B*	T	recessive	Glucose homeostasis	0.220	2.76E-11	Excluded
18	rs4939883	*LIPG*	T	dominant	Lipid metabolism	0.217	5.33E-11	Excluded
19	rs173539	*CETP*	T	dominant	Lipid metabolism	0.231	4.97E-12	Included
20	rs11071657	*C2CD4B*	G	dominant	Glucose homeostasis	0.223	1.30E-11	Excluded
21	rs1800588	*LIPC*	C	dominant	Lipid metabolism	0.234	4.57E-12	Included
22	rs1532085	*ALDH1A2*	G	codominant	Lipid metabolism	0.228	1.28E-11	Excluded
23	rs10938397	*PRDX4P1*	A	recessive	Obesity	0.229	1.12E-11	Excluded

24	rs2191349	*GTF3AP5*	G	recessive	Glucose homeostasis	0.233	1.02E-11	Excluded
25	rs1137101	*LEPR*	G	dominant	Obesity	0.220	3.36E-11	Excluded
26	rs7499892	*CETP*	C	dominant	Lipid metabolism	0.238	2.66E-12	Included
27	rs10946398	*CDKAL1*	A	codominant	Glucose homeostasis	0.230	7.02E-12	Excluded
28	rs6499640	*FTO*	G	dominant	Obesity	0.228	1.26E-11	Excluded
29	rs6265	*BDNF*	T	dominant	Obesity	0.233	3.89E-12	Excluded
30	rs1077834	*LIPC*	T	dominant	Lipid metabolism	0.226	2.26E-11	Excluded
31	rs2070895	*LIPC*	G	dominant	Lipid metabolism	0.226	2.26E-11	Excluded
32	rs2548861	*WWOX*	G	recessive	Lipid metabolism	0.224	2.40E-11	Excluded
33	rs11671664	*GIPR*	A	codominant	Glucose homeostasis	0.246	9.23E-13	Included
34	rs6773957	*ADIPOQ*	G	dominant	Obesity	0.239	2.24E-12	Excluded
35	rs5219	*KCNJ11*	T	dominant	Glucose homeostasis	0.233	6.71E-12	Excluded
36	rs7173964	*C2CD4B*	G	dominant	Glucose homeostasis	0.246	8.14E-13	Included
37	rs5882	*CETP*	A	dominant	Lipid metabolism	0.246	7.60E-13	Included
38	rs1501299	*ADIPOQ*	G	dominant	Obesity	0.246	6.80E-13	Included
39	rs17782313	*MC4R*	T	dominant	Obesity	0.246	8.91E-13	Excluded
40	rs12970134	*MC4R*	G	dominant	Obesity	0.246	8.96E-13	Excluded
41	rs4775041	*ALDH1A2*	G	recessive	Lipid metabolism	0.236	7.85E-12	Excluded
42	rs1861868	*FTO*	C	recessive	Obesity	0.232	8.15E-12	Excluded
43	rs2338104	*KCTD10*	G	dominant	Lipid metabolism	0.253	3.65E-13	Included
44	rs10468017	*ALDH1A2*	T	recessive	Lipid metabolism	0.251	5.27E-13	Excluded
45	rs10906115	*CDC123*	G	dominant	Glucose homeostasis	0.233	8.84E-12	Excluded
46	rs7903146	*TCF7L2*	C	dominant	Glucose homeostasis	0.249	5.62E-13	Excluded
47	rs1558902	*FTO*	A	recessive	Obesity	0.243	1.90E-12	Excluded
48	rs780094	*GCKR*	C	dominant	Glucose homeostasis	0.240	1.40E-12	Excluded
49	rs2867125	*TMEM18*	C	recessive	Obesity	0.242	1.37E-12	Excluded
50	rs340874	*PROX1*	T	codominant	Glucose homeostasis	0.247	6.30E-13	Excluded
51	rs11920090	*SLC2A2*	A	recessive	Glucose homeostasis	0.255	3.29E-13	Included
52	rs9939609	*FTO*	A	recessive	Obesity	0.241	2.31E-12	Excluded
53	rs10885122	*BTBD7P2*	G	recessive	Glucose homeostasis	0.251	9.60E-13	Excluded
54	rs9941349	*FTO*	C	recessive	Obesity	0.246	1.19E-12	Excluded

55	rs10811661	*CDKN2B*	C	recessive	Glucose homeostasis	0.258	2.65E-13	Included
56	rs6548238	*TMEM18*	C	recessive	Obesity	0.245	1.08E-12	Excluded
57	rs2815752	*RPL31P12*	G	recessive	Obesity	0.256	3.15E-13	Excluded
58	rs7944584	*MADD*	T	codominant	Glucose homeostasis	0.252	4.32E-13	Excluded
59	rs4149268	*ABCA1*	C	dominant	Lipid metabolism	0.248	7.53E-13	Excluded
60	rs9989419	*CETP*	A	recessive	Lipid metabolism	0.244	2.23E-12	Excluded
61	rs1801260	*CLOCK*	G	dominant	Obesity	0.236	3.26E-12	Excluded
62	rs660339	*UCP2*	A	codominant	Obesity	0.247	7.92E-13	Excluded
63	rs560887	*G6PC2*	T	dominant	Glucose homeostasis	0.229	1.74E-11	Excluded
64	rs1121980	*FTO*	A	recessive	Obesity	0.241	3.36E-12	Excluded
65	rs925946	*BDNF-AS*	T	recessive	Obesity	0.253	6.30E-13	Excluded
66	rs7034200	*GLIS3*	A	dominant	Glucose homeostasis	0.241	3.77E-12	Excluded
67	rs659366	*UCP2*	T	dominant	Obesity	0.234	4.65E-12	Excluded
