# Supplementary material for: Genetic Background of Metabolically Healthy and Unhealthy Obesity Phenotypes in Hungarian Adult Sample Population
Source: Int J Mol Sci. 2023 Mar 8;24(6):5209. doi: 10.3390/ijms24065209 (PMC10049500; doi:10.3390/ijms24065209)
Supplement: Supplementary file 1 [file ijms-24-05209-s001.zip › Supplementary Table 2.pdf]

International Journal of
Molecular Sciences

Supplementary Table S2. Association of optimized genetic risk score with different parameters associated with the metabolically unhealthy obesity.

	β-value (95%CI)	*p*-value
BMI (kg/m2)	-0.13 (-0.16 – 0.87)	0.871
Waist circumference (cm)	0.27 (-0.31 – 0.37)	0.874
Systolic blood pressure (mmHg)	1.00 (0.34 – 1.65)	0.003*
Diastolic blood pressure (mmHg)	0.24 (-0.12 – 0.60)	0.184
Fasting triglycerides level (mmol/L)	0.07 (0.01 – 0.13)	0.024*
Fasting HDL-C level (mmol/L)	-0.01 (-0.02 – 0.01)	0.084
Fasting glucose level (mmol/L)	0.08 (-0.01 – 0.15)	0.032*
Fasting insulin (mU/L)	0.96 (-0.06 – 1.99)	0.066
HOMA-IR	0.07 (0.01 – 0.14)	0.016*
CRP (mg/L)	-0.03 (-0.20 – 0.15)	0.770

*: $p < 0.05$, **: significance threshold determined after Bonferroni test correction: $p < 0.0026$.

Note: Multivariate linear regression models were adjusted for age, sex, BMI (except in the case where BMI was the outcome variable), education, anti-hypertensive, antidiabetic, and lipid-lowering treatment. 95%CI: 95% confidence intervals; BMI: body mass index; HDL-C: high-density lipoprotein cholesterol; HOMA-IR: homeostasis model assessment of insulin resistance; CRP: C-reactive protein.
